# Exceptionally simple, rapidly replaced teeth in sauropod dinosaurs demonstrate a novel evolutionary strategy for herbivory in Late Jurassic ecosystems

**DOI:** 10.1186/s12862-021-01932-4

**Published:** 2021-11-06

**Authors:** Keegan M. Melstrom, Luis M. Chiappe, Nathan D. Smith

**Affiliations:** The Dinosaur Institute, Natural History Museum of Los Angeles County, 900 W Exposition Blvd, Los Angeles, CA USA

**Keywords:** Dental complexity, Diet, Dinosauria, Macroevolution, Tooth

## Abstract

**Background:**

Dinosaurs dominated terrestrial environments for over 100 million years due in part to innovative feeding strategies. Although a range of dental adaptations was present in Late Jurassic dinosaurs, it is unclear whether dinosaur ecosystems exhibited patterns of tooth disparity and dietary correlation similar to those of modern amniotes, in which carnivores possess simple teeth and herbivores exhibit complex dentitions. To investigate these patterns, we quantified dental shape in Late Jurassic dinosaurs to test relationships between diet and dental complexity.

**Results:**

Here, we show that Late Jurassic dinosaurs exhibited a disparity of dental complexities on par with those of modern saurians. Theropods possess relatively simple teeth, in spite of the range of morphologies tested, and is consistent with their inferred carnivorous habits. Ornithischians, in contrast, have complex dentitions, corresponding to herbivorous habits. The dentitions of macronarian sauropods are similar to some ornithischians and living herbivorous squamates but slightly more complex than other sauropods. In particular, all diplodocoid sauropods investigated possess remarkably simple teeth. The existence of simple teeth in diplodocoids, however, contrasts with the pattern observed in nearly all known herbivores (living or extinct).

**Conclusions:**

Sauropod dinosaurs exhibit a novel approach to herbivory not yet observed in other amniotes. We demonstrate that sauropod tooth complexity is related to tooth replacement rate rather than diet, which contrasts with the results from mammals and saurians. This relationship is unique to the sauropod clade, with ornithischians and theropods displaying the patterns observed in other groups. The decoupling of herbivory and tooth complexity paired with a correlation between complexity and replacement rate demonstrates a novel evolutionary strategy for plant consumption in sauropod dinosaurs.

**Supplementary Information:**

The online version contains supplementary material available at 10.1186/s12862-021-01932-4.

## Background

Extinct dinosaurs exhibit a remarkable variety of dental morphologies ranging from narrow, peg-like teeth to tightly-packed, elaborate dental batteries, which are thought to directly reflect diet [1]. Despite nearly two centuries of study and the discovery of novel tooth forms, our understanding of dinosaur dietary ecologies remains limited compared to other groups, such as mammals [1, 2]. Much of what is known relies on osteological comparisons to extant analogues, frequently noted in early discoveries [3], microwear analyses [2, 4–9], and the rare preservation of stomach contents [10–14]. The few studies that have applied quantitative methods to the study of dinosaur dentitions have yielded tantalizing insights. For example, an investigation of theropod dental disparity linked diet to the survival of neornithine birds after the end-Cretaceous mass extinction [15]. Dental microwear analyses of sauropods strongly suggest that different clades employed differing feeding strategies, which may have permitted these large-bodied animals to live in the same environment [4, 16]. Another common quantitative dental assessment is the reconstruction of tooth replacement patterns and rates (all toothed dinosaurs exhibit polyphyodonty) [2, 17–26]. Histological investigations demonstrate that dental replacement rates vary substantially across the dinosaur clade, from every 777 days in the theropod *Tyrannosaurus* to 14 days in the sauropod *Nigersaurus* [2, 17, 19].

Dental topographic methods have yielded key insights into both modern and past ecosystems. For instance, using a quantitative measure of dental complexity—orientation patch count rotated (OPCR)—Evans et al. [27] demonstrated that phenotypic tooth complexity is related to diet in living carnivorans and rodents, with carnivores possessing simple teeth and herbivores displaying complex teeth. Using patterns of dental complexity, Wilson et al. [28] hypothesized that multituberculates ecologically radiated approximately 20 million years prior to the end-Cretaceous mass extinction. To a lesser extent, these methods have also been applied to dentigerous saurians (i.e. lepidosaurs and crocodilians), demonstrating that the relationship between diet and dental complexity originally observed in mammals is found across the amniote clade, in spite of major differences in development and replacement patterns [29]. Despite their popularity and ubiquity, similar quantitative assessments of dental morphology have not been applied to dinosaurs, leaving broad questions unaddressed. For example, were dinosaurian ecosystems characterized by the same range of tooth complexity seen in modern saurian communities, and did dinosaurs exhibit similar correlations between diet and dental complexity as observed in modern amniotes?

**Fig. 1:**
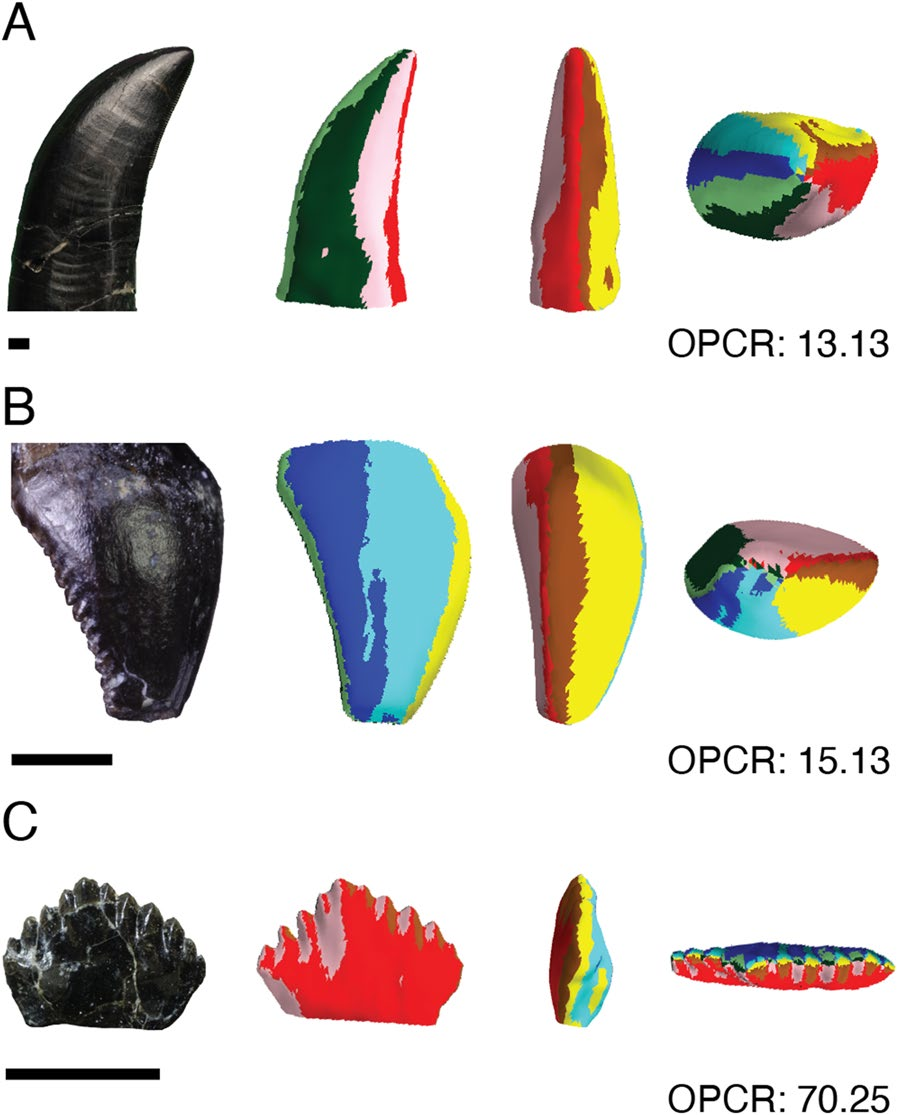
Dental morphology and OPCR maps of select Late Jurassic dinosaurs. **A** *Allosaurus* (Photo: UMNH VP 5841; OPCR model: BYU 759) **B** *Gargoyleosaurus* (DMNH EPV0.27,726) and **C** *Nanosaurus* (MWC 5822) from the Upper Jurassic Morrison Formation. Dental morphology varies considerably between dinosaurs that lived during the Late Jurassic and OPCR allows for a numerical representation of differing shapes. Note that some details, such as the serrations of *Allosaurus*, are not detected in OPCR analyses. Dental complexity maps in labial (left centre), distal (right centre), and occlusal (right) view. Models shown have 10,000 faces and were measured at a minimum of three triangles per patch. Scale bar equals 0.25 cm. *BYU* Brigham Young University, *DMNH* Denver Museum of Nature and Science, *MWC* Museum of Western Colorado, *UMNH* Utah Museum of Natural History

The Late Jurassic represents an excellent system for testing hypotheses regarding dinosaur dentition. The paleoenvironment and taxic diversity of both fauna and flora are relatively well known, in part due to an extensive history of worldwide collection, but especially in that of western North America [30]. During this epoch, dinosaurs attained a remarkable diversity, with theropods, sauropods, and ornithischians exhibiting a wide array of taxa and ecologies. In particular, the herbivorous sauropods achieved colossal sizes and an exceptional diversity, with many coexisting diplodocoids and macronarians. In spite of broad similarities in the sauropod bauplan, these two clades are characterized by marked morphological differences in both cranial and postcranial elements. Importantly, these skeletons sometimes include nearly complete cranial material with associated dental elements, which reveal a diverse range of tooth shapes, especially amongst herbivores [19, 31–34] (Fig. 1). The combination of skeletal and dental microwear differences strongly suggest a partitioning of plant resources. The abundance of dentigerous cranial material has also enabled the estimation of tooth replacement rates across the sauropod and theropod clade [19, 20, 22, 35]. These data, combined with the taxonomic diversity and range of dental morphologies, present an exceptional opportunity to test ecosystem-wide patterns during a relatively narrow time span.

Here, we seek to improve our understanding of dinosaur dietary ecology by testing two hypotheses using quantitative morphological assessments. First, we evaluate the relationship between diet and dental complexity in Late Jurassic dinosaurs using the OPCR method, specifically testing whether carnivores possessed simple teeth and herbivores exhibited complex dentitions. Second, we test the hypothesis that dental complexity values are related to tooth replacement rate. Our results shed light on key evolutionary innovations that allowed multiple herbivorous dinosaur clades to diversify and coexist.

## Results

### Dental complexity

**Fig. 2:**
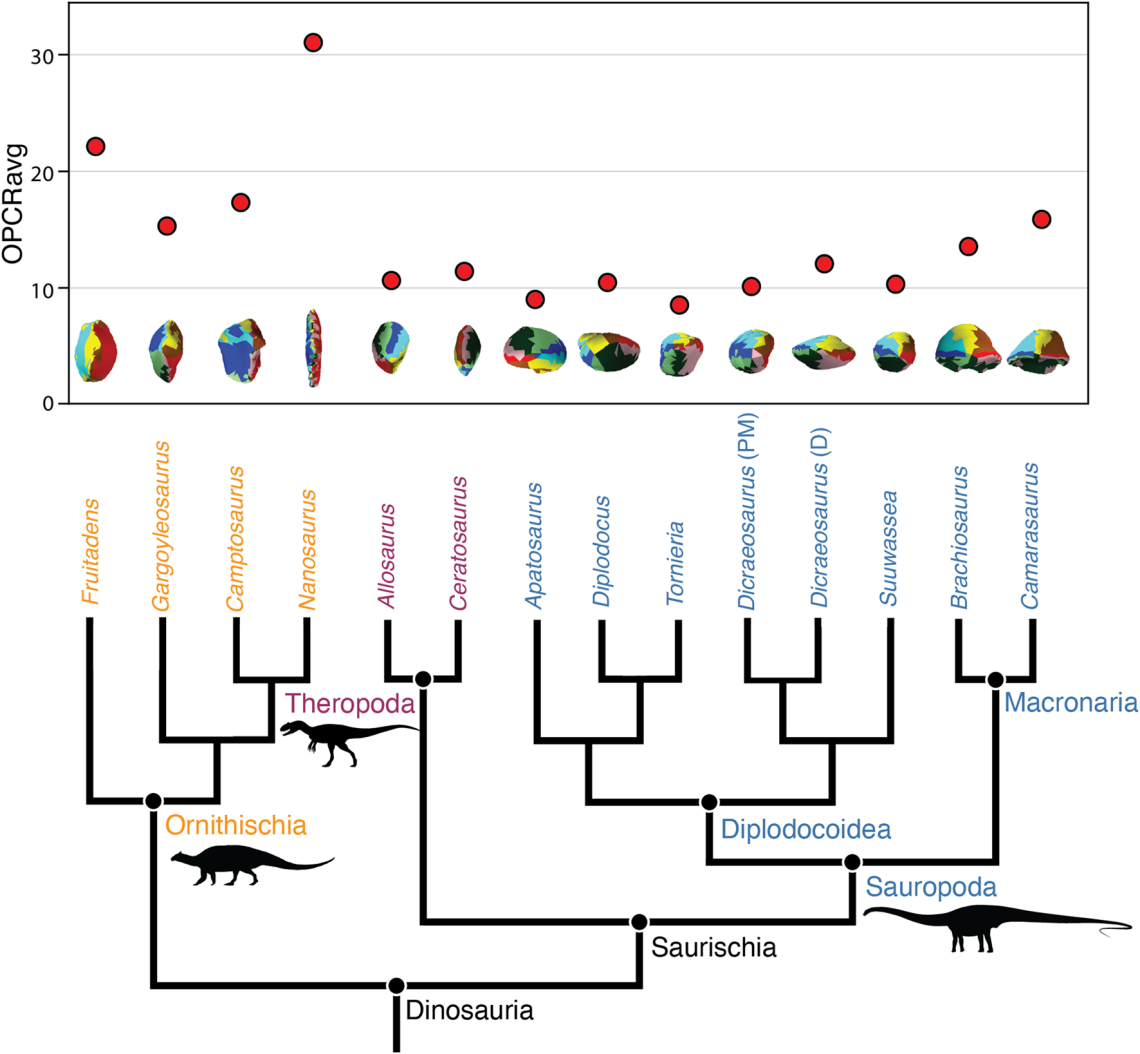
Average dental complexity and phylogenetic relationships of Late Jurassic dinosaurs. Ornithischian herbivores (orange, left) typically have higher dental complexities (3 patch analyses) than both theropods (purple, centre) and sauropods (blue, right). Herbivorous diplodocoid sauropods exhibit tooth complexities similar to theropod carnivorous theropods, which contrasts with patterns observed in extant and extinct herbivores. Illustrations of dental complexity in occlusal view are shown below OPCR values. *D* dentary, *PM* premaxilla and maxilla. Silhouettes courtesy of S. Abramowicz and phylopic.org

**Fig. 3:**
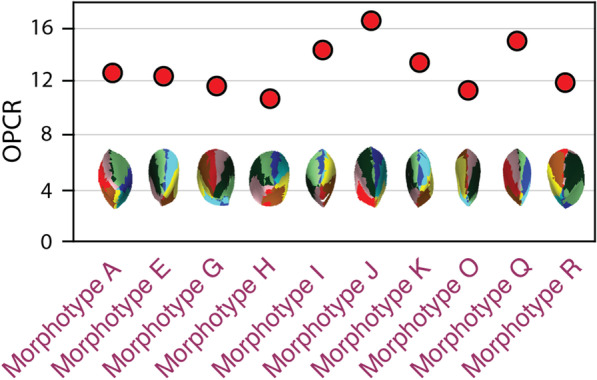
Dental complexity of theropod tooth morphotypes. Theropods from the Late Jurassic exhibit a wide range of shapes and this is reflected in differences in dental complexity. Complexity maps illustrating dental morphologies in occlusal view are below dental complexity values

Late Jurassic dinosaurs exhibit a wide range of dental complexities (Fig. 1). Average tooth complexity varies between 9.03 and 33.75 patches per tooth (PPT) when measured with a minimum polygon count threshold of three triangles (3 patch) and a model size of 1000 triangles (Figs. 2 and 3; Additional file 1, Additional file 1: Table S2). At a minimum polygon count threshold of five triangles (5 patch) and a model size of 1000 triangles, dental complexity patterns are consistent, but dental disparity decreases, with a range from 8.60 to 24 patches (Additional file 1: Table S2). This range is similar to those of extant saurians when measured using the same procedures despite differences in gross surface morphology [36].

**Fig. 4:**
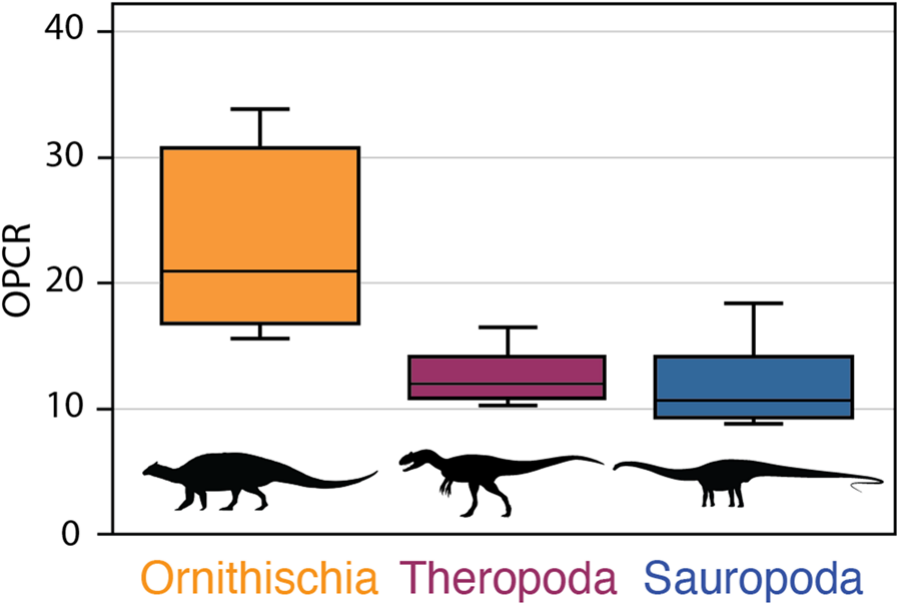
Dental complexity of the three dinosaur clades: ornithischians, theropods, and sauropods. Theropods are characterized by uniformly low dental complexities, similar to extant carnivorous saurians. Sauropods, the largest herbivores to ever walk the Earth, also possess simple teeth, in stark contrast to living herbivores. Ornithischians exhibit much higher tooth complexities, which resemble the patterns observed in modern herbivorous amniotes. Data are derived from average dental complexity of each genus. For box and whisker plots, the median is designated by a horizontal line, boxes encompass 25–75 % quartiles, whereas upper and lower quartiles are shown with vertical lines (i.e. whiskers). Silhouettes courtesy of S. Abramowicz and phylopic.org

Our results demonstrate dental complexities differ between the three major dinosaur clades when measuring the average value of each genus (Kruskal-Wallis: *P* < 0.01). Pairwise comparisons of 3 patch analyses show no significant difference between mean, median, and distribution of theropod and sauropod dental complexities (Student’s t-test; Mann-Whitney *U*; Kolmogorov–Smirnov test: *P* > 0.05, 9999 replicates) (Fig. 4). The mean, median, and distribution of theropod and sauropod tooth complexity are both significantly different from ornithischians (Student’s t-test, Mann-Whitney *U*, and Kolmogorov–Smirnov: *P* < 0.01, 9999 replicates). When the individual tooth complexities of macronarian and diplodocoid sauropods are analysed separately there is a significant difference between mean dental complexity of these groups (Student’s t-test: *P* < 0.01, t = 10.23, 9999 replicates). Macronarians in our sample are characterized by significantly higher complexities (14.28 PPT, 3 patch) than the narrow-crowned teeth of diplodocoids (9.74 PPT, 3 patch), as well as a greater range in complexities. The mean, median, and distribution of both sauropod clades are significantly different from theropods and ornithischians (Student’s t-test, Mann-Whitney *U*, and Kolmogorov–Smirnov: *P* < 0.01, 9999 replicates). The dental complexities of saurischians and ornithischians are significantly different for all statistical tests (Student’s t-test, Mann-Whitney *U*, and Kolmogorov–Smirnov: *P* < 0.01, 9999 replicates). At 5 patch analyses, results are similar to previous analyses with no significant difference between saurischian clades (Student’s t-test, Mann-Whitney *U*: *P* > 0.05). Surface complexity of measured theropods varied between 10.0 and 16.5 PPT (3 patch) when measured at a resolution of 1000 triangles (Figs. 2 and 3). In some cases (e.g. Morphotypes A and K), minor cracks or damaged enamel caused an increase in complexity. In other tooth morphotypes (O, Q, and R) the narrow cutting edge increased complexity by being broken up periodically, resulting in high complexities relative to other carnivorous saurians [36].

**Fig. 5:**
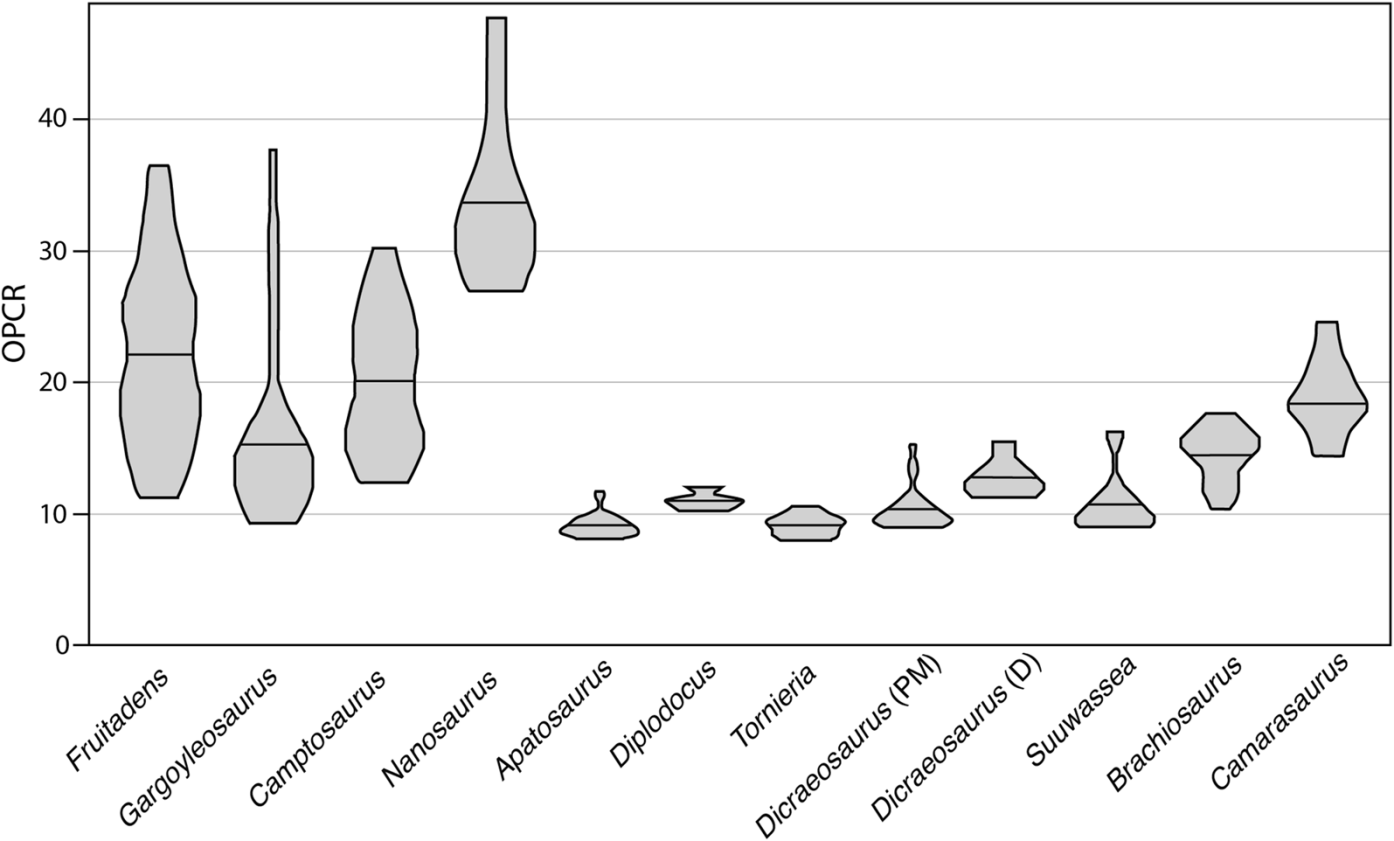
Violin plot illustrating the range in dental complexity of herbivorous dinosaurs. The width of each bar represents the relative proportion of teeth with a particular complexity and the median is designated by a horizontal line. Sauropods have a much lower range in complexities compared to ornithischians, the latter of which are frequently characterized by a heterodont dentition. All analyses were performed at models with 1000 triangles and a minimum patch count of three polygons

The seven measured sauropod genera display a greater range of dental complexities than those of theropods (Figs. 2 and 4; Additional file 1, Additional file 1: Table S2). The distribution of sauropod dental complexities is somewhat bimodal, with the broad-crowned teeth of macronarians exhibiting higher complexities than the narrow-crowned teeth of diplodocoids. The lowest value measured for a Late Jurassic dinosaur occurs in *Tornieria* (8 patches), a diplodocid from Tanzania [37]. Other diplodocids (e.g. *Apatosaurus*, *Diplodocus*) also possess relatively simple teeth, between 9 and 11 PPT (3 patch) with dental models of 1000 triangles (Figs. 2, 4 and 5). These sauropods are characterized by narrow-crowned, peg-like teeth. The two macronarians included in this study, *Brachiosaurus* and *Camarasaurus*, exhibit the highest average dental complexities of our sauropod sample, 14.39 and 18.38 PPT (3 patch), respectively (Figs. 2 and 5). The teeth of these dinosaurs are characterized by a convex labial margin, concave lingual margin, and rugose wrinkled enamel [18]. In both groups, tooth wear, including the development of large facets or the loss of enamel wrinkles, does not significantly impact dental complexity.

Ornithischians possess the most complex teeth of sampled Late Jurassic dinosaurs (Figs. 1, 2 and 4; Additional file 1). *Nanosaurus* is characterized by labiolingually compressed teeth with up to ten cusps (Additional file 1: Fig. S1C) and exhibits an average complexity of 33.75 PPT (3 patch) at a model size of 1,000 triangles. *Fruitadens*, one of the smallest known non-avian dinosaurs [38], possesses an average dental complexity of 23.63 PPT at the same resolution. Unworn teeth of *Fruitadens* can reach up to 36.5 PPT at the same parameters, rivalling the complexities of herbivorous iguanids [36]. Other ornithischians have lower complexity values. *Camptosaurus* has an average complexity of 20.15 PPT (3 patch) when measured with a model of 1000 triangles. *Gargoyleosaurus*, an early ankylosaur, possesses simpler teeth, with a complexity value of 15.68 PPT when measured at the same resolution. Similar to *Fruitadens*, erupted and worn teeth are simpler than unworn dentitions. In one *Camptosaurus* specimen (MWC 2), noteworthy ridges and mesiodistal cusps are worn away, simplifying dental morphology and complexity values of this specimen, although wear facets of UMNH VP 16455 do not result in simplified complexities. This simplification contrasts with sauropods, in which tooth wear generally does not dramatically alter dental morphology and complexity.

Across measured dinosaurs, phylogenetic relationships exert less of an influence on OPCR values then predicted under Brownian motion, with K, a measure of phylogenetic signal, less than 1, although phylogenetic effects are still significant (Blomberg’s *K* = 0.82, *P* = 0.019) [39]. Similarly, Pagel’s lambda is approximately 1 (λ = 1.04, *P* < 0.01), suggesting that OPCR values between taxa vary in proportion to their shared phylogenetic history. When measuring sauropods only, there is a significant phylogenetic signal in OPCR values, beyond what would be expected by a Brownian motion model of character evolution (Blomberg’s *K* = 1.62, *P* = 0.012). Within Sauropoda there is more variance in dental complexity between clades (i.e. diplodocoids and macronarians) than within them, suggesting that OPCR, at least in sauropods, is associated with phylogenetic relationships. In this case, diplodocoids possess simpler teeth whereas macronarians exhibit more complex dentitions than would be expected if phylogeny were not playing a role. In this reduced dataset, Pagel’s lambda is greater than 1, but this result is marginally non-significant (λ = 1.21, *P* = 0.059).

### Replacement rate

To better understand patterns of tooth shape in Late Jurassic dinosaurs, we tested for a correlation between replacement rate (measured in days) and dental complexity [2, 19, 20]. Across all sampled specimens, there is a positive correlation but it is not statistically significant (Pearson’s product-moment correlation: t = 1.99, *P* = 0.072, r = 0.51; Kendall’s rank correlation τ: z = 1.69, *P* = 0.09, τ = 0.37). For these data, however, replacement rates have been estimated for ornithischians based on the average of known replacement rates (96 days), which is likely an underestimate [17, 40]. When measuring sauropods only, with both replacement rates of *Dicraeosaurus* taken into account [20], there is a significant positive relationship between dental complexity and replacement rate (Pearson’s product-moment correlation: t = 2.97, *P* = 0.03; r = 0.77; Kendall’s rank correlation τ: z = 2.37, *P* = 0.02, τ = 0.69).

We then used PGLS and phylogenetic ANOVA to investigate patterns between OPCR and dental replacement rate in a phylogenetic framework. PGLS analyses recover differing results for different datasets. Tests of the complete dinosaur dataset do not recover replacement rate as a significant predictor of OPCR in either 3 or 5 patch datasets using both Brownian motion and Ornstein–Uhlenbeck models (*P* > 0.05), whereas the Pagel’s “lambda” model does result in a significant predictive relationship (*P* = 0.01; *P* = 0.02 for 3 and 5 patch datasets, respectively). A phylogenetic ANOVA found a non-significant relationship in both OPCR datasets (*P* > 0.05). When investigating only saurischians, in which tooth replacement data are better known, the replacement rate is a significant predictor of OPCR under Brownian motion (*P* = 0.02 for both 3 and 5 patch datasets), but not Ornstein–Uhlenbeck, Pagel’s “lambda”, or a phylogenetic ANOVA (*P* > 0.05). This contrasts with the results of sauropods only, in which dental complexity is not a function of replacement rate under Brownian motion, Pagel’s “lambda”, and phylogenetic ANOVA (*P* > 0.05), but there is a positive, significant relationship with the Ornstein-Uhlenbeck model of evolution (3 patch dataset: *P* = 0.04). In sauropod-only analyses, if *Dicraeosaurus* is treated as two separate taxa, because of the two replacement rates for upper and lower dentitions, all PGLS and phylogenetic ANOVA return a significant (*P* < 0.05) positive relationship between tooth complexity and replacement rate for 3 and 5 patch datasets.

## Discussion

**Fig. 6:**
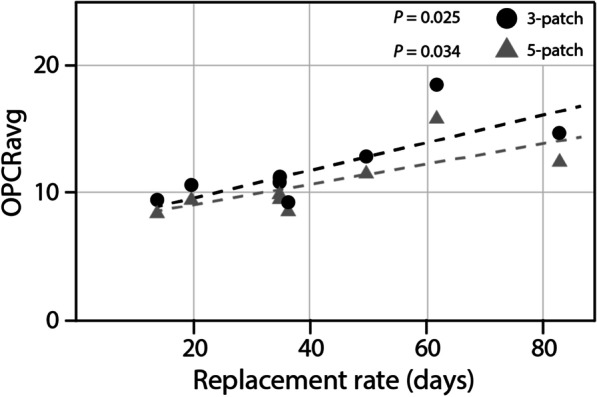
The relationship between dental replacement rate and average tooth complexity in sampled Late Jurassic sauropod dinosaurs. Simpler teeth (lower OPCRavg) are associated with shorter replacement rates, whereas more complex dentitions tend to have longer replacement rates. This association is supported in both three and five count minimum polygon thresholds and when phylogenetic relationships are taken into account

Here, we demonstrate a unique relationship between phenotypic tooth complexity and dietary ecology in Late Jurassic dinosaurs. Because independent evidence of general feeding ecology (e.g. herbivory, carnivory) is known for a variety of dinosaur taxa, diets of these clades can be reconstructed with relative confidence allowing for the testing of relationships between diet and dental complexity. In measured ornithischians and theropods, the previously recovered pattern is maintained; carnivorous theropods have simple teeth, whereas herbivorous ornithischians display the most complex dentitions in sampled dinosaurs (Figs. 1, 2, 4 and 5). Ornithischians, like extant herbivorous saurians [29], express the greatest range of complexities (Fig. 4), reflecting their heterodont condition. In both living saurians and mammals, there is a positive relationship between dental complexity and the amount of plant matter consumed [27, 29, 41]. Hypercarnivores and carnivores (i.e. animals that primarily consume animal material but also small amounts of plants) possess simple teeth (i.e. low dental complexity). Herbivores exhibit the highest dental complexity values, reflecting additional cusps, crenulations, and grinding surfaces that enlarge the dental surface area and help break down plant material prior to chemical digestion. Herbivorous sauropods, however, complicate this otherwise straightforward pattern by possessing relatively simple dentitions. In fact, with their extremely simple teeth, diplodocoid sauropods appear to exhibit a novel strategy of plant consumption (Figs. 2, 5 and 6). Therefore, our hypothesis that dinosaurs that lived during the Late Jurassic exhibit modern patterns of dental complexity and diet is falsified, at least for the diplodocoids included in this study.

In spite of noteworthy differences in morphology, the dental complexity of diplodocoids closely resembles that of carnivorous theropods, and in some cases is even simpler. The narrow peg-like teeth of this group lack prominent cusps that add complexity to the teeth of other herbivores, both living and extinct [29, 42]. Sauropod herbivory is not in doubt (but see Stokes [43]), as the group is characterized by a number of features that are related to this diet, including large body size and wide rib cages [44]. The limited role these teeth play in food processing combined with the absence of precise dental occlusion may be expected to result in simpler dentitions in diplodocoids, but these characteristics also are observed in many groups of extant lepidosaurs that are typified by complex dentitions. Together, this suggests that sauropods, especially diplodocoids, are unique in currently sampled taxa, living and extinct, in having low dental complexity values associated with an herbivorous diet.

To investigate this pattern further, we looked at the tooth replacement rates of sauropods [2, 19–21, 45]. Replacement rates of dinosaurs are calculated by counting the differences in incremental lines of von Ebner (previously shown to be deposited daily in living crocodilians) between successive tooth generations [17]. There is a marked disparity in dinosaur tooth replacement rates. For example, the narrow peg-like teeth of *Diplodocus* were replaced at a relatively rapid rate, each tooth every 35 days approximately, whereas the replacement rate is slower in macronarians, which often exhibit a wider and spatulate morphology (e.g. 62 days for *Camarasaurus*) [19]. In herbivorous taxa with well-documented replacement rates, there is a clear correlation between dental complexity and tooth replacement rate (r = 0.77; τ = 0.69; *P* < 0.05; Fig. 6). Sauropods with rapid replacement rates tend to have simpler teeth, whereas those that have slower rates possess more complex dentitions (Fig. 6). Analyses that take phylogeny into account demonstrate that this relationship is significant in sauropods. Diplodocoids have the lowest OPCR values of herbivorous saurischians and the fastest rates of tooth replacement. Macronarian sauropods, in contrast, exhibit higher average complexities, longer replacement rates, thicker enamel, and a greater tooth volume [19]. Interestingly, the pattern between complexity and replacement rate appears to extend even to a single taxon. The premaxillary and maxillary teeth of *Dicraeosaurus* were replaced at a faster rate than those of the dentary, and there is a significant difference in complexity of those elements (10.32 and 12.73 PPT, respectively; *P* < 0.01). Unfortunately, precise replacement rate data remain unknown for the ornithischians included in this study, but if they are similar to that of sampled relatives (e.g. *Edmontonia*, *Triceratops*, *Maiasaura*) they are longer than sauropods (279–381 days for adult taxa), which would also be consistent with the distinct correlation between higher dental complexity and slower replacement rate [17].

Narrow, peg-like teeth evolved independently in more than one sauropod lineage [19, 46]. After the disappearance of diplodocids at the beginning of the Early Cretaceous, surviving macronarians developed dentitions that resemble those of diplodocoids (i.e. a similar slenderness index) [46]. In particular, titanosaurs, such as *Antarctosaurus*, *Nemegtosaurus*, and *Rapetosaurus*, developed extremely narrow tooth crowns [47]. Coupled with this change in morphology was a notable increase in the number of replacement teeth (e.g. Museo Provincial Carlos Ameghino, MPCA-79), an indication of high replacement rates, although titanosaur replacement rates remain undocumented histologically [19, 46, 47]. The independent evolution of narrow-crowned teeth strongly implies a simple complexity in titanosaurs. If coupled with high replacement rates (as is hypothesized in MPCA-79 [19]), this may indicate convergence in herbivory strategies in distantly related sauropod clades. Thus, diplodocoids, and potentially titanosaurs, may have evolved a method of plant consumption and processing not yet documented in either extinct or extant amniotes. Instead of complex teeth that break down plant material, these sauropods utilized extremely simple teeth with narrow bands of enamel that were quickly worn and rapidly replaced. This style of herbivory may have been greatly beneficial because it had a dual advantage for a long-necked plant-eater: enabling resource partitioning with contemporaneous herbivores (other sauropods, ornithischians, and additional amniotes) while also allowing for a lightened skull [19]. Additionally, simple-toothed herbivores independently occur in the Late Jurassic and Late Cretaceous. There was a remarkable shift in plant community composition during this time interval, from gymnosperm-dominated communities to those in which angiosperms were the principal floral component of terrestrial environments [48]. Thus, the presence of this morphology in both environments suggests this approach is not associated with a particular dietary specialization but instead represents an adaptation for a broader feeding strategy.

Previous research interpreted these morphological specializations with low-browsing feeding. The diet of diplodocoids with narrow-crowned teeth has been hypothesized to largely consist of abrasive plants, although the exact food differs [4, 46, 49–51]. An analysis of snout shape and microwear of diplodocoids found evidence for multiple feeding strategies, ranging from low to medium browse and both selective and non-selective diets [4]. The plant genus *Equisetum* (i.e. horsetail) was suggested to be an excellent potential food source in the Morrison Formation environment, in fact it yields energy levels that surpass extant grasses, but a noteworthy downside is that its surfaces are rich in silica [50, 52]. Simple teeth that are rapidly replaced due to extensive wear may be an adaptation to an exceptionally abrasive diet [50], but this stands in stark contrast to the complex dentitions found in the vast majority of herbivorous mammals, squamates, and extinct crocodyliforms [27, 29, 41, 42]. Complex teeth provide a greater surface area that breaks down plant material prior to ingestion, facilitating the absorption of nutrients. The evolution of a more abrasive diet and the concurrent change in dental complexity has previously been investigated in equids [53]. In equids, as abrasive foods became a greater proportion of diet and body size increased, dental complexity grew through the addition of increasingly fine features [53]. Here, we show the opposite trend in large-bodied taxa that continuously replaced their teeth. Instead of high volume, complex dentitions, browsing sauropods develop simple teeth that were rapidly worn and subsequently replaced. Simple and narrow dentitions, with a lower enamel and dentin volume [19], that could be rapidly replaced would have permitted the consumption of abrasive plant material that may otherwise have been detrimental to taxa with a more permanent dentition. Simultaneously, smaller teeth would have been advantageous to a retaining a light skull and long neck, which has been hypothesized to be a key factor in sauropod gigantism and success [54]. Interestingly, simple teeth are also known in two species of living herbivorous squamates, the skinks *Egernia stokesii* and *Tiliqua rugosa*, but these taxa are closely related to non-herbivorous species [29, 55]. Additionally, replacement rates from skinks remain unknown, hindering our ability to test for the pattern between simple teeth and rapid replacement rates in living herbivorous saurians.

In contrast to the novel patterns observed in sauropods, theropod dental complexity is consistent with that of other carnivorous saurians. This dental simplicity of sampled theropods reinforces previous evidence, including serrations and sharp cusps, that support the view of carnivory across this group during the Late Jurassic. We sampled 12 tooth morphotypes and found that theropods display the lowest disparity of sampled clades in spite of the diverse collection of morphologies exhibited (Fig. 3). The majority of theropod teeth are relatively simple, approximately 10–12 PPT for 3 patch analyses. In part, this is due to fine features, such as serrations, not being detected by OPCR analyses. Although these structures are critical for the consumption of animal material, they are lost during model preparation that permit comparisons between different sized taxa or scanners [36]. Finer morphological elements were also not detected in previous analyses of both extant saurians and extinct crocodyliforms and so this absence is not unique to theropod dinosaurs [29, 42].

Despite the overall low OPCR values of carnivorous theropods, there is a noteworthy range in complexities (i.e. 8.25–16.5 PPT) (Fig. 5). This result demonstrates that dental complexity differences reflect, at least to some degree, the morphological disparity that existed in Late Jurassic theropods. In living saurians, the carnivore dietary category encompasses a broad range of diets [29]. The range in morphology and complexity, especially when coupled with body size variation, likely reflects a similar pattern with theropods, with taxa potentially specializing on different faunal resources (i.e. niche partitioning) [56]. The long-held inference of a uniform diet across theropods of the Late Jurassic should be tested with greater rigor, as the substantial disparity in dental size, shape, and complexity may reflect an otherwise unacknowledged ecological range in this diverse group. In particular, the variation in tooth complexity of theropods present during the narrow temporal range of the Late Jurassic may be a precursor of the later ecological divergence found in Cretaceous theropods. Studies of extant avians show that ecological transitions to omnivory from carnivory are much more common than directly from carnivory to herbivory [57]. Therefore, although Late Jurassic theropods in general possessed simple teeth, the variation in qualitative features, as well as complexity, observed in this clade likely reflect a dietary diversity already in place prior to the more pronounced ecological shifts that occur during the Cretaceous.

The radiation of theropods into novel ecological roles also coincided with numerous shifts in cranial and dental morphology [58]. Although some groups, such as therizinosaurs, evolve heterodonty and even multi-cusped teeth that superficially resemble the morphology of extant iguanids [59], many others possess simplified, conical-shaped teeth [58]. In some cases, herbivorous habits are associated with tooth loss in theropods [58]. This initial simplification and subsequent loss of teeth, especially in the distal jaw, may represent a similar adaptation towards herbivory as that of diplodocoids. Although this remains untested, it would demonstrate an intriguing pattern in which multiple dinosaur clades independently simplified dentitions for an herbivorous lifestyle, which would stand in stark contrast to the patterns observed in extant herbivores, saurian and mammal alike.

## Conclusions

This work demonstrates the remarkable potential of quantitative dental analyses to illuminate dinosaurian palaeoecology. In particular, OPCR analyses have the capacity to address key macroevolutionary questions, measure and describe patterns within and across dinosaur communities around the world, and even allow for direct comparison to modern faunas. Here, we find clear patterns between dinosaur dental complexity and diet. In some cases, carnivorous theropods possess simple teeth and herbivorous ornithischians display more complex tooth shapes, reflecting broad patterns observed in both living and extinct amniotes. Surprisingly, theropods display a wide dental disparity, despite overall low values of tooth complexity. This variation may not only be related to taxonomic diversity but also to dietary differences among Late Jurassic theropods that have remained otherwise undetected. This ecological disparity may provide early clues to the dietary breadth that would characterize the later evolution of this clade during the Cretaceous. In the case of sauropods, however, the widely documented pattern between tooth complexity and diet breaks down, representing a novel strategy of herbivory. Dental complexity is correlated to tooth replacement rate in sauropod dinosaurs, even when phylogeny is taken into account. When combined with other morphological features of the diplodocoid clade, this strategy likely represents an adaptation to massive body sizes and living alongside a diverse fauna of megaherbivores (Additional file 2).

## Methods

### Specimens and sampling

We investigated dental complexities from a broad range of Late Jurassic dinosaurs (Additional file 1: Fig. S1, Table S1). This dataset includes four ornithischians, seven sauropods, two theropods and ten theropod tooth morphotypes, capturing much of the dental disparity of Late Jurassic dinosaurs. We measured a combination of isolated teeth and those preserved within jaws, the latter comprising the majority of the dataset. Although the majority of our dataset comprises specimens from the Morrison Formation of western North America, we include key samples from Europe and Africa that fill in important gaps in species diversity and dental morphologies [60]. Following previous work, we sample teeth along the entire dentigerous element [29, 42].

To model the influence of phylogenetic relationships, we built a time-calibrated phylogenetic tree based on a combination of large, cladistic datasets that analysed broad relationships between major dinosaur groups [61] and those that focused on specific clades [62–64]. While phylogenetic uncertainty and some conflict exist between studies of dinosaur relationships, the phylogenetic positions of taxa sampled in this study are in broad agreement across recent studies. Branch lengths exert considerable influence on character evolution [65] and unfortunately no definitive time-calibrated phylogeny exists for non-avian dinosaurs. To calibrate our tree, we used estimates based on data presented in Benson et al. [61], as well as values from Whitlock [62] and D’Emic [63] for sauropod relationships. We accounted for differences in the timing of clade origination by manually modifying branch lengths to characterize the effect of different estimates, which did not result in noteworthy differences in statistical analyses. All taxa included in this study are from a relatively narrow temporal range; the ages of the Morrison Formation and the Middle Dinosaur Member of the Tendaguru Formation were estimated to be 152 MA (the Kimmeridgian-Tithonian boundary) based on a narrow range of ages estimated from microfossil and ^40^Ar/^39^Ar dating [66, 67]. Theropod tooth morphotypes were not included in phylogenetic analyses. Phylogenies that were used in statistical analyses (see below) were written in Newick format in R and generated using the *read.tree* function from the ‘ape’ package (version 5.4-1) [68, 69].

We generated 3D models of dinosaur dentition from a combination of microCT and CT scans. CT data of sauropods, theropods, and *Fruitadens* were taken from previously published sources, whereas *Camptosaurus, Gargoyleosaurus*, and *Nanosaurus* were scanned at the University of Southern California Molecular Imaging Center. Individual scanning parameters are available in their original publications and the Additional file (Additional file 1: Table S1). We generated 3D models of both worn and unworn dentitions in Avizo Lite (Version 2020.1; Thermo Fisher Scientific). Following procedures outlined by previous research on mammal and saurian dentitions [36, 70], we standardized surfaces to 10,000 triangles (± 1 triangles) using MeshLab’s [71] ‘Simplification: Quadratic Edge Collapse Decimation’ tool. The dentitions were then smoothed using the Laplacian smooth function with three steps followed by a second down-sample to 1000 triangles. Dental complexity measurements can be influenced by factors not directly related to surface morphology, including tooth size, digitization method, and number of triangles in each model. Larger teeth produce higher resolution digital models, which, in turn, contain a greater amount of morphological information, a result of more triangles in each model. Thus, larger teeth and models with a greater number of triangles are more likely to result in a higher OPCR value. Similarly, CT scans will likely yield lower OPCR values than microCT scans if scanning the same specimen because the latter captures more information per unit area. Standardizing dental models to a consistent resolution/triangle count largely removes these effects as well as additional minor damage that may have occurred during the fossilization process [36, 70]. In some cases, additional morphological information can be lost during this standardization process, but previous work has demonstrated this did not significantly alter broad complexity patterns [36]. 3D dental models used in this research are available for download at https://www.morphosource.org/projects/000345163.

### OPCR and statistical analysis

We measured tooth complexity using the 3D orientation patch count rotated (OPCR) method, which quantitatively assesses dental morphology and allows for teeth with no homologous landmarks to be directly compared [27, 28, 72]. This method assigns a cardinal or ordinal direction to each triangle, groups contiguous triangles with the same orientation together into patches, and then sums the patches. Following this, models are rotated 5.625° and patch counts are recalculated. This latter step is repeated seven times, with the final complexity measurement averaged over a total of eight trials. This method provides a numerical representation of surface shape that is robust to minor differences in model orientation. In sampled living animals, dental complexity tends to be simple in carnivores and complex in herbivores, with dietary generalists frequently falling between the two end-members [27, 29, 41]. We used MorphoTester, a free open-source dental topology software, to measure the dental complexity of each tooth [72]. This program, written originally in the Python programming language, analyses full 3D polygon meshes. This contrasts with a previous application of OPCR, which utilizes Surfer Manipulator and raster-based digital elevation models [27–29, 73]. These models differ from 3D polygon meshes in that they associate X and Y values with only a single Z value (i.e. 2.5D) and these two sets of OPCR data are not directly comparable [72]. Surfer Manipulator captures topography of the occlusal margin, the dental surface that interacts with food, whereas MorphoTester measures the entire dental surface. This additional measurement by MorphoTester systematically results in higher complexity measurements relative to Surfer Manipulator [36, 72], however, MorphoTester is freely available and is better able to batch process large numbers of data files. PLY files were uploaded to MorphoTester and evaluated using a minimum patch size of three and five triangles to facilitate comparisons with previous work [72]. Results that utilize minimum patch counts of three triangles capture finer details, such as small cusps, but are also more likely to detect dental damage, whereas minimum patch counts of five triangles are more robust to these issues, but may not identify fine details. We report both sets of results below (Additional file 1: Table S2, Additional file 2). Following previous research on saurians, we averaged the complexity of all teeth for a single taxon (OPCRavg).

We performed statistical analyses in RStudio (version 1.3.1073; R version 4.0.2) and PAST4 [74, 75]. Replacement rates were taken from previous studies on sauropods [19–21, 35, 45] and theropods [22]. The dental replacement rate of *Suuwassea* was estimated based on the average rate of its closest relative *Dicraeosaurus*, whereas *Apatosaurus* was estimated based on a similar tooth replacement count between these specimens and *Nigersaurus* [19, 21]. The significance of the correlation between dental complexity and tooth replacement rate was evaluated using Pearson’s product-moment correlation and Kendall’s rank correlation τ by the *cor.test* function from the ‘stats’ package (version 4.0). We tested for phylogenetic signal and its significance in OPCR and replacement rate results using the K-statistic and Pagel’s lambda, which were calculated using the *phylosig* function from the ‘phytools’ package (version 0.7-47) [76]. We used multiple methods implemented in R to assess the relationship between replacement rate and dental complexity in a phylogenetic framework. A phylogenetic ANOVA was preformed using the *procD.pgls* function (SS.type = I, effect.type = F, iter = 9999) from the package ‘geomorph’ (version 3.3.1), in which average dental complexity of each genus was the response variable and replacement rate in days was the independent variable [77]. We also ran phylogenetic generalized least squares (PGLS) using the ‘ape’ package and its dependencies [69, 78] (version 5.4-1). We first created correlation structures using Pagel’s lambda (*corPagel*), Brownian motion (*corBrownian*), and Ornstein–Uhlenbeck (*corMartins*) models of trait covariance [68, 79]. Following this, we used the *gls* function (‘nlme’ package, version 3.1–148 [80]) to perform generalized least squares analysis where dental complexity was a function of replacement rate [68].

## Supplementary Information

**Additional file 1: Supplemental method explainations. Fig. S1.** Dental complexity of the major dinosaur groups, with sauropods broken into their two primary clades included in this study: diplodocoids and macronarians. . **Table S1.** Information on the Late Jurassic dinosaurs measured. **Table S2.** Average dental complexity values of Late Jurassic dinosaurs.

**Additional file 2.** OPCR values for each genus and theropod morphotype.

## Data Availability

3D models dental models used in this study are available on https://www.morphosource.org/projects/000345163.
