# Supplementary material for: Exceptionally simple, rapidly replaced teeth in sauropod dinosaurs demonstrate a novel evolutionary strategy for herbivory in Late Jurassic ecosystems
Source: BMC Ecol Evol. 2021 Nov 6;21:202. doi: 10.1186/s12862-021-01932-4 (PMC8571970; doi:10.1186/s12862-021-01932-4)
Supplement: Supplementary file 1 — Additional file 1: Supplemental method explainations. Fig. S1. Dental complexity of the major dinosaur groups, with sauropods broken into their two primary clades included in this study: diplodocoids and macronarians. . Table S1. Information on the Late Jurassic dinosaurs measured. Table S2. Average dental complexity values of Late Jurassic dinosaurs. [file 12862_2021_1932_MOESM1_ESM.docx]

Additional file 1 for Exceptionally simple, rapidly replaced teeth in sauropod dinosaurs demonstrate a novel evolutionary strategy for herbivory in Late Jurassic ecosystems

Keegan M. Melstrom, Luis M. Chiappe, Nathan D. Smith

In this study, we measure all teeth preserved in jaw elements. Topographic studies of mammalian dentitions frequently only sample premolars and molars [1–6], but establishing homologous teeth throughout Sauria (i.e. archosaurs and lepidosaurs) is difficult, especially because many taxa are effectively homodont. Sampled dinosaurs are a mix of morphologically homodont (e.g. *Allosaurus*, *Diplodocus*) and heterodont (e.g. *Fruitadens*) taxa, making the selection of premolariform and molariform teeth potentially inconsistent. To facilitate reproducibility and avoid arbitrary tooth selection when sampling different specimens, previous research on saurians measured teeth from the entire tooth row [7–11]. Additionally, investigations of lepidosaur dental patterns demonstrated no significant difference between the teeth of different jaw elements [7, 10]. We measured both isolated teeth and those preserved *in situ* within their respective dentigerous elements, although the latter was considered preferable. Isolated teeth were only measured for theropod morphotypes and *Camarasaurus* (UMNH VP 5873). Lastly, for teeth preserved within jaw elements, we measure both erupted and unerupted teeth, so long as the morphology resembles unworn adult teeth. In sauropods, this permitted the measuring of up to the second generation of unerupted teeth. Together, the combination of erupted and unerupted, as well as worn and unworn, dentitions allows for the full range of morphologies exhibited by individuals of the same genus to be sampled in our investigation.

*Scan Parameters*

We CT scanned *Camptosaurus*, *Gargoyleosaurus*, and *Nanosaurus*, at the University of Southern California Molecular Imaging Center. Two jaws of *Camptosaurus* (MWC 2) were scanned at a voxel size of 0.089 mm^3^ and 0.095 mm^3^ and a pixel resolution of 1044 x 1124 and 1057 x 1027 for 2000 slices. UMNH VP 16455 was scanned at a voxel size of 0.077 mm^3^ and pixel resolution of 1160 x 1193 for 2000 slices. *Nanosaurus* was microCT scanned at a voxel resolution of 0.026 mm^3^ and pixel resolution of 1749 x 1782. *Gargoyleosaurus* was CT scanned in two session and stitched together. Both scans had a total of 1442 slices at a pixel resolution of 2000 x 2000 and a voxel size of 0.123 mm^3^.

*Mesh generation and preparation*

   To generate 3D models, we segmented dentigerous material in Avizo Lite (Version 2020.1; Thermo Fisher Scientific), using the ‘Segmentation Editor’ to highlight the tooth crown and a portion of the root. We sampled erupted teeth, but also unerupted elements when they were well-developed. The inclusion of both erupted and unerupted teeth allowed us to analyse a range of tooth wear as well as a larger dataset. In the case of diplodocoid sauropods, the most developed two dental generations were used, whereas we used only the first dental generation for macronarian sauropods and ornithischians. Differences in morphology and reduced density contrast in developing teeth excluded additional tooth generations. Erupted and sampled unerupted teeth do not differ in complexity unless erupted teeth are worn (e.g. *Fruitadens*). Teeth that had experienced minor damage (i.e. missing pieces or characterized by cracks) were digitally repaired in Avizo Lite. Following this, teeth were oriented in Avizo Lite with the occlusal surface parallel with the z-axis [1]. Following segmentation, we exported each tooth as an individual surface PLY file.

         Tooth scans were then opened and edited in MeshLab [12] (Version 2016.12). Portions of the model belonging to the tooth root were removed. Topographic analyses that use vector-based triangulated polygon meshes are sensitive to differences in mesh face count [13]. To account for this, we standardized surfaces to 10,000 faces (+/- 1 face) using MeshLab’s ‘Simplification: Quadratic Edge Collapse Decimation’ tool following the recommendations of previous work [14]. After the first down-sample, dentitions were smoothed using the Laplacian smooth function with three steps. This step reduces variation associated with filling tooth cracks and errors caused by generating 3D models from surfaces that directly contacted one another. Smoothing was done following down-sampling because it otherwise could lead to surface artefacts that would affect subsequent analyses. These steps can result in the loss of genuine details (e.g. the disappearance of serrations in Fig. 1A or small cusps in Fig. 1B), but also eliminate issues related to differences in scan source, resolution, and tooth damage. Tooth size may also impact scan quality (i.e., larger teeth will have a greater number of detectable features), but holding the number of triangles per model at a standard value reduces the impact of size. In spite of the down-sampling, most major morphological features continued to be detected. Lastly, models were down-sampled to 1,000 faces. In some cases, original models were composed of fewer than 10,000 faces and were only down-sampled to 1,000 faces. Although 1,000 face models possess less information than 10,000 faces, they still preserve much of the information detected in analyses and are appropriate for subsequent OPCR analyses. 3D models used in this research are available for download at morphosource.org.


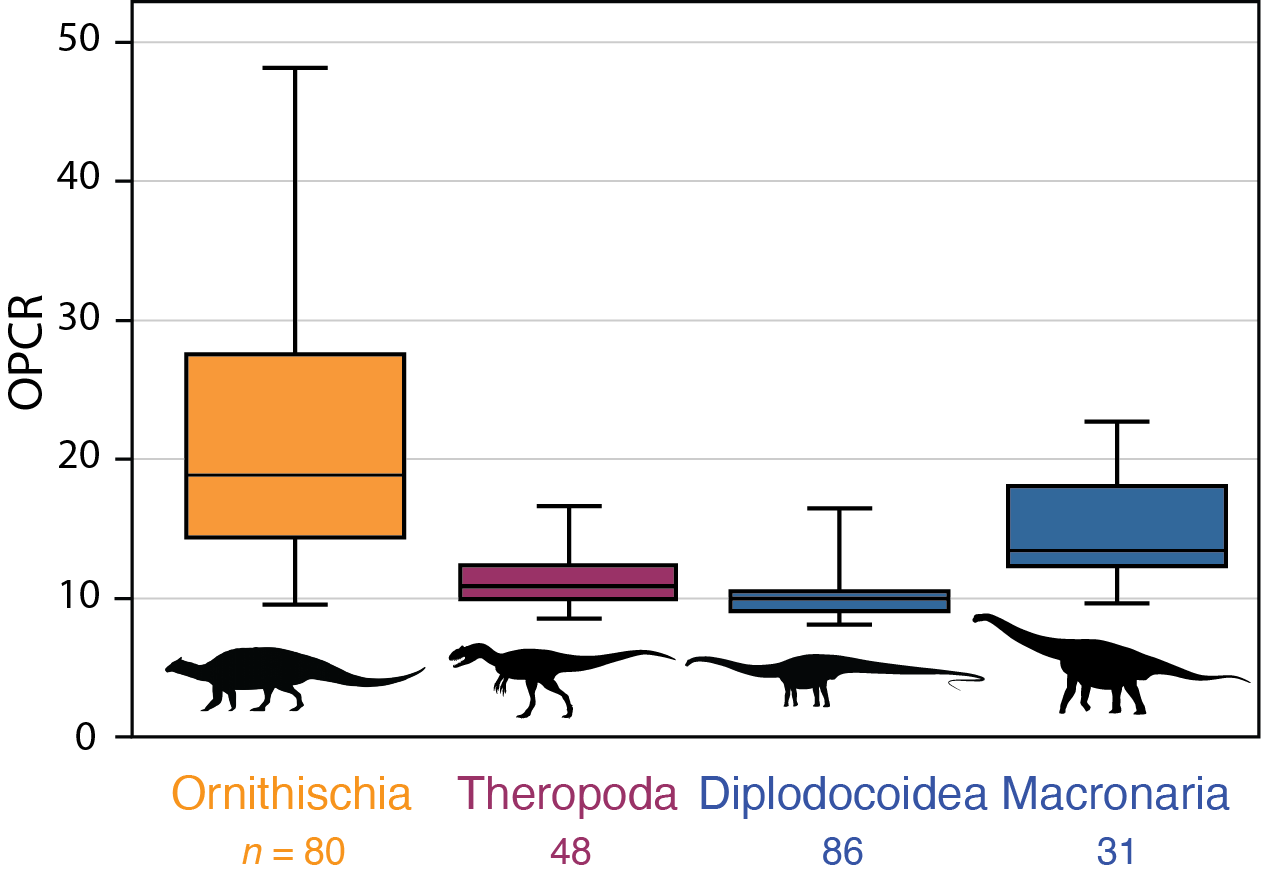


Fig. S1. Dental complexity of the major dinosaur groups, with sauropods broken into their two primary clades included in this study: diplodocoids and macronarians. Data are derived from individual tooth analyses with *n* representing the number of teeth measured for each clade. Silhouettes courtesy of S. Abramowicz and phylopic.org.

Table S1. Information on the Late Jurassic dinosaurs measured. Abbreviations: ANS, Academy of Natural Sciences, Philadelphia; BYU, Brigham Young University; CM, Carnegie Museum of Natural History; DFMMh/FV: Dinosaurier-Freilichtmuseum Münchehagen/Verein zur Förderung der Niedersächsischen Paläontologie; DMNH, Denver Museum of Nature and Science; GZG, Geowissenschaftliches Zentrum der Universität Göttingen; LACM, Natural History Museum of Los Angeles County; MB.R., Museum für Naturkunde Berlin; NLMH, Niedersächsisches Landesmuseum; MWC, Museum of Western Colorado; UMNH, Utah Museum of Natural History; USNM, National Museum of Natural History.

| Taxon/Morphotype | Specimen number | Number of teeth measured | Formation | Region | Scan Source |
| --- | --- | --- | --- | --- | --- |
| *Allosaurus* | BYU 759, UMNH VP C-173 | 33 | Morrison | Western U.S. | [15] |
| *Apatosaurus* | MWC 6002, MWC 8430 | 29 | Morrison | Western U.S. | [16] |
| *Brachiosaurus* | USNM 5730 | 21 | Morrison | Western U.S. | [17] |
| *Camarasaurus* | UMNH VP 5527, UMNH VP 5873 | 10 | Morrison | Western U.S. | [18] |
| *Camptosaurus* | MWC 2, UMNH VP 16455 | 20 | Morrison | Western U.S. | This publication |
| *Ceratosaurus* | BYU 12893 | 5 | Morrison | Western U.S. | [15] |
| *Dicraeosaurus* | MB.R.2337, MB.R.2338, MB.R.2339 | 31 | Tendaguru | Southeast Tanzania | [19] |
| *Diplodocus* | CM 11255 | 5 | Morrison | Western U.S. | [18] |
| *Fruitadens* | LACM 11574, LACM 128258 | 21 | Morrison | Western U.S. | [20] |
| *Gargoyleosaurus* | DMNH EPV.27726 | 28 | Morrison | Western U.S. | This publication |
| *Nanosaurus* | MWC 5822 | 11 | Morrison | Western U.S. | This publication |
| *Suuwassea* | ANS 21122 | 8 | Morrison | Western U.S. | J. Whitlock (unpublished data) |
| *Tornieria* | MB.R.2343, MB.R.2345, MB.R.2347 | 19 | Tendaguru | Southeast Tanzania | [21] |
| Theropod morphotype A | MB.R.2800 | 1 | “Purbeck/ Serpulit” | Northern Germany | [22] |
| Theropod morphotype E | GZG. V.010.379 | 1 | Lindener Berg Hannover | Northern Germany | [22] |
| Theropod morphotype G | NLMH101378a | 1 | “Tönniesberg Coll. Struckmann” | Northern Germany | [22] |
| Theropod morphotype H | GZG.V.010.373 | 1 | Lindener Berg Hannover | Northern Germany | [22] |
| Theropod morphotype I | GZG.V.010.327 | 1 | Lindener Berg Hannover | Northern Germany | [22] |
| Theropod morphotype J | NLMH 106235b | 1 | Tönniesberg Coll. Dahl | Northern Germany | [22] |
| Theropod morphotype K | GZG.V.010.334 | 1 | Kahleberg b. Echte | Northern Germany | [22] |
| Theropod morphotype O | DFMMh/ FV 530 | 1 | Bed 83, Langenberg Quarry, Malm Group | Northern Germany | [22] |
| Theropod morphotype Q | DFMMh/ FV 383 | 1 | Bed 83, Langenberg Quarry, Malm Group | Northern Germany | [22] |
| Theropod morphotype R | DFMMh/ FV 790.5 | 1 | Bed 83, Langenberg Quarry, Malm Group | Northern Germany | [22] |

Table S2. Average dental complexity values of Late Jurassic dinosaurs. The parameters of the dental analyses are contained in the parentheses. The minimum patch size is represented by the numerator and total triangle count of each tooth model is represented by the denominator. Abbreviations: D, dentary; PM, premaxilla and maxilla.

| Taxon/Morphotype | OPCRavg (3/1000) | OPCRavg (5/1000) | OPCRavg (3/10000) | OPCRavg (5/10000) |
| --- | --- | --- | --- | --- |
| *Allosaurus* | 10.63 | 9.50 | 13.00 | 10.98 |
| *Apatosaurus* | 9.12 | 8.50 | 17.03 | 11.93 |
| *Brachiosaurus* | 14.39 | 12.38 | 17.56 | 14.98 |
| *Camarasaurus* | 18.39 | 15.84 | 19.90 | 16.34 |
| *Camptosaurus* | 20.15 | 15.51 | 35.71 | 27.12 |
| *Ceratosaurus* | 10.90 | 9.85 | 14.05 | 12.20 |
| *Dicraeosaurus* (PM) | 10.37 | 9.71 | 11.81 | 9.82 |
| *Dicraeosaurus* (D) | 12.73 | 11.52 | 11.85 | 10.23 |
| *Diplodocus* | 11.00 | 10.18 | 9.75 | 8.90 |
| *Fruitadens* | 22.01 | 16.63 | 42.10 | 32.62 |
| *Gargoyleosaurus* | 15.68 | 13.48 | 21.39 | 17.20 |
| *Nanosaurus* | 33.75 | 24.00 | 52.68 | 43.82 |
| *Suuwassea* | 10.64 | 9.61 | 10.11 | 9.30 |
| *Tornieria* | 9.15 | 8.68 | 10.84 | 9.19 |
| Theropod morphotype A | 12.50 | 11.38 | 21.63 | 18.00 |
| Theropod morphotype E | 12.25 | 10.63 | 19.75 | 16.38 |
| Theropod morphotype G | 11.625 | 9.00 | 29.63 | 23.63 |
| Theropod morphotype H | 10.50 | 9.63 | 28.88 | 20.13 |
| Theropod morphotype I | 14.25 | 13.00 | 24.38 | 17.63 |
| Theropod morphotype J | 16.50 | 13.75 | 29.25 | 23.75 |
| Theropod morphotype K | 13.375 | 11.50 | 45.75 | 29.75 |
| Theropod morphotype O | 11.25 | 9.25 | 30.38 | 25.00 |
| Theropod morphotype Q | 15.00 | 10.63 | 41.00 | 31.00 |
| Theropod morphotype R | 11.875 | 10.13 | 30.88 | 21.75 |

**Additional file 1 References**

1. Evans AR, Wilson GP, Fortelius M, Jernvall J. High-level similarity of dentitions in carnivorans and rodents. Nature. 2007;445:78–81.

2. Wilson GP, Evans AR, Corfe IJ, Smits PD, Fortelius M, Jernvall J. Adaptive radiation of multituberculate mammals before the extinction of dinosaurs. Nature. 2012;483:457–60.

3. Pineda-Munoz S, Lazagabaster IA, Alroy J, Evans AR. Inferring diet from dental morphology in terrestrial mammals. Methods Ecol Evol. 2016;8:481–491.

4. Selig KR, Sargis EJ, Silcox MT. The frugivorous insectivores? Functional morphological analysis of molar topography for inferring diet in extant treeshrews (Scandentia). J Mammal. 2019;100:1901–1917.

5. Selig KR, Khalid W, Silcox MT. Mammalian molar complexity follows simple, predictable patterns. Proc Natl Acad Sci U S A. 2020;118:e2008850118.

6. López-Torres S, Selig KR, Prufrock KA, Lin D, Silcox MT. Dental topographic analysis of paromomyid (Plesiadapiformes, Primates) cheek teeth: more than 15 million years of changing surfaces and shifting ecologies. Hist Biol. 2018;30:76–88. doi:10.1080/08912963.2017.1289378.

7. Melstrom KM. The relationship between diet and tooth complexity in living dentigerous saurians. J Morphol. 2017;278:500–522.

8. Melstrom KM, Irmis RB. Repeated evolution of herbivorous crocodyliforms during the age of dinosaurs. Curr Biol. 2019;29:2389–2395.

9. Melstrom KM, Wistort ZP. Quantification conundrum: Just how repeatable are dental complexity measurement methods? In: Integrative and comparative biology. 2019. p. E370.

10. Christensen K, Melstrom KM. Quantitative analyses of squamate dentition demonstrate novel morphological patterns. PLoS One. 2021;16:e0257427.

11. Melstrom KM, Wistort Z. Accepted. The application of dental complexity metrics on extant saurians. Herpetologica.

12. Cignoni P, Callieri M, Corsini M, Dellepiane M, Ganovelli F, Ranzuglia G. MeshLab: An open-source mesh processing tool. Eurographics Ital Chapter Conf. 2008;:129–136.

13. Berthaume MA, Winchester J, Kupczik K. Effects of cropping, smoothing, triangle count, and mesh resolution on 6 dental topographic metrics. PLoS One. 2019;14:e0216229.

14. Pampush JD, Spradley JP, Morse PE, Harrington AR, Allen KL, Boyer DM, et al. Wear and its effects on dental topography measures in howling monkeys (*Alouatta palliata*). Am J Phys Anthropol. 2016;161:705–721.

15. D’Emic MD, O’Connor PM, Pascucci TR, Gavras JN, Mardakhayava E, Lund EK. Evolution of high tooth replacement rates in theropod dinosaurs. PLoS One. 2019;14:e0224734.

16. McHugh JB. Evidence for niche partitioning among ground-height browsing sauropods from the Upper Jurassic Morrison Formation of North America. Geol Intermt West. 2018;5:95–103.

17. D’Emic MD, Carrano MT. Redescription of brachiosaurid sauropod dinosaur material from the Upper Jurassic Morrison Formation, Colorado, USA. Anat Rec. 2020;303:732–758.

18. D’Emic MD, Whitlock JA, Smith KM, Fisher DC, Wilson JA. Evolution of high tooth replacement rates in sauropod dinosaurs. PLoS One. 2013;8:e69235.

19. Schwarz D, Kosch JCD, Fritsch G, Hildebrandt T. Dentition and tooth replacement of *Dicraeosaurus hansemanni* (Dinosauria, Sauropoda, Diplodocoidea) from the Tendaguru Formation of Tanzania. J Vertebr Paleontol. 2015;35:e1008134.

20. Butler RJ, Galton PM, Porro LB, Chiappe LM, Henderson DM, Erickson GM. Lower limits of ornithischian dinosaur body size inferred from a new Upper Jurassic heterodontosaurid from North America. Proc R Soc B Biol Sci. 2010;277:375–381.

21. Sattler F. Tooth replacement of the sauropod dinosaur *Tornieria africana* (Fraas) from Tendaguru (Late Jurassic,Tanzania). 2014.

22. Gerke O, Wings O. Multivariate and cladistic analyses of isolated teeth reveal sympatry of theropod dinosaurs in the Late Jurassic of Northern Germany. PLoS One. 2016;11:e0158334.
